# The role of graft T-cell size in patients receiving alemtuzumab serotherapy for non-malignant disorders: results of an institutional protocol

**DOI:** 10.1038/s41598-023-50416-6

**Published:** 2024-01-10

**Authors:** Ambreen Pandrowala, Sanna Khan, Darshan Kataria, Manasa Kakunje, Varsha Mishra, Dhruv Mamtora, Sangeeta Mudaliar, Minnie Bodhanwala, Bharat Agarwal, Prashant Hiwarkar

**Affiliations:** 1 Department of Blood and Marrow Transplantation, Bai Jerbai Wadia Hospital for Children, Mumbai, 400012 India; 2 Department of Pathology, Bai Jerbai Wadia Hospital for Children, Mumbai, India; 3 Department of Paediatrics, Bai Jerbai Wadia Hospital for Children, Mumbai, India; 4 Department of Paediatric Haematology, Bai Jerbai Wadia Hospital for Children, Mumbai, India

**Keywords:** Bone marrow transplantation, Lymphocytes, Translational immunology, Transplant immunology, Allotransplantation

## Abstract

Although graft T cells assist in engraftment, mediate antiviral immune-reconstitution, and cause graft-versus-host disease, graft size is not determined by T-cell content of the graft. The conventional method of graft size determination based on CD34+ cells with alemtuzumab serotherapy is associated with delayed immune reconstitution, contributing to an increased risk of viral infections and graft failure. Alemtuzumab, a long half-life anti-CD52 monoclonal antibody is a robust T-cell depleting serotherapy, and relatively spares memory-effector T cells compared to naïve T cells. We therefore hypothesized that graft size based on T-cell content in patients receiving peripheral blood stem cell graft with alemtuzumab serotherapy would facilitate immune-reconstitution without increasing the risk of graft-versus-host disease. We retrospectively analysed twenty-six consecutive patients with non-malignant disorders grafted using alemtuzumab serotherapy and capping of graft T cells to a maximum of 600 million/kg. The graft T-cell capping protocol resulted in early immune-reconstitution without increasing the risk of severe graft-versus-host disease. Graft T-cell content correlated with CD4+ T-cell reconstitution and acute graft-versus-host disease. The course of CMV viraemia was predictable without recurrence and associated with early T-cell recovery. No patient developed chronic graft-versus-host disease. Overall survival at one year was 100% and disease-free survival was 96% at a median of 899 days (range: 243–1562). Graft size determined by peripheral blood stem cell graft T-cell content in patients receiving alemtuzumab serotherapy for non-malignant disorders is safe and leads to early T-cell immune-reconstitution with excellent survival outcomes.

## Introduction

An ideal transplant protocol should have low regimen-related toxicity and a low incidence of graft-versus-host disease (GvHD) with early immune-reconstitution to prevent viral disease. Stable mixed chimerism can reverse the phenotype in non-malignant disorders (NMDs) and full donor chimerism is not a pre-requisite. Reduced-toxicity conditioning regimen has been successfully used to prevent chemotherapy-related toxicity in NMDs. Immunoablation can be performed using anti-thymocyte globulin (ATG), a polyclonal antibody, or alemtuzumab, a monoclonal antibody against CD52 in transplant protocols employing serotherapy for GVHD prophylaxis.

Alemtuzumab is a robust T-cell depleting agent, and in addition to the T and B-cell depletion, alemtuzumab depletes dendritic cells which are important in the pathophysiology of gut GvHD^1^. The main concern with alemtuzumab is a prolonged terminal half-life of 2–3 weeks^2^, resulting in depletion of graft T cells. Excessive depletion of graft T cells delays T-cell reconstitution and increases the risk of viral infection^3^. High alemtuzumab levels on day zero leads to excessive depletion of graft T cells and increased risk of graft failure^4^.

In a phase 2 trial of individualised ATG dosing, ATG dosing algorithm was derived on the basis of body weight, absolute lymphocyte count of the recipient before transplant conditioning, and the stem cell source^5^. The study showed improvement in CD4+ T-cell reconstitution compared to the historical cohort without increasing the incidence of GvHD and graft failure. In the context of alemtuzumab, different strategies have been used to optimize the use of alemtuzumab including early or distal administration (day-22 or earlier), late or proximal dosing schedule (day-12 or later), pharmacokinetic modeling to predict day 0 dosing, etc^6–12^. However, no strategy has been shown to effectively improve immune-reconstitution without increasing the risk of GvHD and graft failure.

Although graft T cells assist in engraftment, mediate antiviral immune-reconstitution, and cause GvHD, graft size is not determined by T-cell content of the graft. The conventional method of graft size determination based on CD34+ cells allow grafting with a maximum of 200–300 million T cells^13^. To enhance the immune-reconstitution, our institutional practice has evolved to use PBSC grafts with T-cell capping and alemtuzumab serotherapy for patients with NMDs undergoing transplant from HLA-matched related or unrelated donors. Alemtuzumab preferentially depletes naïve T cells and thus relatively spares the memory-effector T cells^14–16^. This has been observed in the clinic and in a non-human primate model^14^. We, therefore hypothesized that capping with a high dose of T cells in patients receiving PBSC graft with alemtuzumab serotherapy would facilitate memory-effector reconstitution without increasing the risk of GvHD.

## Materials and methods

In the retrospective analysis, all patients ≤ 18 years receiving HLA-matched family donor (10/10) and HLA-matched unrelated donor (10/10) or HLA-matched related or unrelated donor (9/10 bidirectional or ≤ 2 HLA antigens in GvH direction) from March 2019 to October 2022 were included.

Alemtuzumab was used for immunoablation. Grafts from HLA-matched family donors (10/10) were categorized as low-risk GvHD grafts, while all other grafts were classified as high-risk GvHD grafts. A total dose of 0.5 mg/kg and 0.9 mg/kg was administered from day-6 to day-4 for low-risk and high-risk GvHD grafts, respectively.

Myeloablation was performed in accordance with internationally accepted protocols. GvHD prophylaxis consisted of ciclosporin for 6 months followed by tapering over 6 weeks with trough levels of 100–150 μg/L, combined with mycophenolate mofetil for the first 28 days followed by taper over the following 7 days. However, early taper or cessation of immunosuppression was considered for mixed chimerism, viral reactivation, serious infection or thrombotic microangiopathy.

PBSCs were used with the capping of T cells up to a maximum of 600 million/kg without CD34+ cell dose capping. Basic lymphocyte subset analysis was evaluated in all patients by flow cytometry at 2, 3, 6, and 9 months as previously described^17^, and in the case of CMV reactivation, immune-reconstitution was performed at the time of onset, and the resolution of viraemia. Extended lymphocyte subset analysis (including recent thymic emigrants) was also performed in 4 patients as previously described^17^. CMV and EBV PCR were monitored in blood once a week and Adenovirus PCR was monitored in both blood and stool samples every week. Chimerism analysis was done by Single Tandem Repeats in peripheral blood or bone marrow at 1, 2, 3, 6, and 12 months or more frequently in cases of mixed chimerism. Mixed chimerism was defined as ≥ 5% recipient cells.

### Outcomes

The effect of T-cell capping on CD4+ T-cell count was studied. The primary outcome measures were CD4+ immune-reconstitution defined as a 1) CD4+ T-cell count of 50/µL at 90 days (± 10) and CD4+ T-cell count of 200/µL at 180 days (± 10). Early CD4+ immune reconstitution was chosen as a primary outcome measure as this was found to be a reliable predictor (in different centers and transplantation settings) for transplantation outcomes such as overall survival, non-relapse mortality, viral reactivation, and GvHD^5,18^. CD4+ T-cell count of 200/µL at 6 months was chosen because CD4+ T-cell count of 200/µL is a set threshold in our institute for relaxing isolation precautions.

Secondary outcome measures included acute and chronic GvHD, viral reactivations (of cytomegalovirus, adenovirus, Epstein Barr virus), overall survival, event-free survival, treatment-related mortality, relapse of primary disease, and graft failure. GvHD and its relation with the dose of alemtuzumab and the T-cell dose were also studied. CMV reactivation and T-cell immune-reconstitution at the onset and resolution of CMV were studied.

Overall survival was defined as the time from transplantation to the last follow-up or death. Event-free survival was defined as survival from HSCT to last contact or an event defined as graft failure, relapse of disease, or death. All surviving patients were censored at the date of the last contact. Treatment-related mortality was defined as causes other than relapse of the disease. Acute GvHD (grade 1–4) was classified according to the Glucksberg criteria^19^ and chronic GVHD (moderate—severe vs no—mild) was classified according to the National Institutes of Health criteria^20^. Graft failure was defined as non-engraftment or graft rejection (i.e. secondary and total loss of donor chimerism). Non-engraftment was defined as failure to reach recovery of neutrophils 60 days after HSCT and engraftment was defined as a neutrophil count greater than 500/µL with or without the use of granulocyte colony-stimulating factor within 40 days.

The study was approved by the Bai Jerbai Wadia Hospital ethics committee (BJWHC/AP/2023/013-V1). All work was carried out in compliance with Ethical Principles for Medical Research Involving Human Subjects outlined in the Helsinki Declaration in 1975. Informed consent was obtained from all patients or their legal guardians.

### Statistical analysis

Incidence of GvHD in patients with 0.5 mg/kg versus 0.9 mg/kg and in patients receiving ≥ 500 million/kg T cells versus < 500 million/kg T cells is shown as bar plots. Chi-square test was used for comparing the two groups.

Spearman correlation between graft T-cell content and CD4+ T cells at 90 days was performed using Graphpad Prism 9.

The probabilities of event-free survival were calculated using the Kaplan–Meier method.

## Results

### Patient and transplant characteristics

Twenty-six consecutive patients (primary immunodeficiencies = 13; benign hematology disorders = 13) transplanted with alemtuzumab serotherapy and T-cell capping were included. The demographics, conditioning regimens, and donor characteristics are mentioned in Table 1.

**Table 1:** Patient characteristics.

No of patients	26
No of transplants	26
Median age of transplant	5 years (6 months-17.1 years)
Female:Male	8:18
**Pretransplant diagnosis**	
Idiopathic severe aplastic anemia Thalassemia Inherited marrow failure syndrome Congenital amegakaryocytic thrombocytopenia Hemophagocytic lymphohistiocytosis Wiskott-Aldrich Syndrome DOCK8 deficiency CD40 ligand deficiency Severe Combined Immunodeficiency CTLA4 deficiency Leukocyte Adhesion Deficiency type 1 CSF2RA deficiency Severe congenital neutropenia	5 4 2 2 3 2 2 1 1 1 1 1 1
**Stem cell source**	
Peripheral blood	26
**Lansky score**	
100 90 80 < = 70	20 1 1 4
Conditioning regimens*	
Fludarabine-Cyclophosphamide Fludarabine-Treosulfan Fludarabine-Treosulfan-Thiotepa Fludarabine	2 8 15 1
**Alemtuzumab from day-6 to day-4**	
0.5 mg/kg 0.9 mg/kg	14 12
**GvHD prophylaxis**	
Ciclosporin and mycophenolate mofetil	26
**Donor Characteristics**	
10/10 HLA-matched Family donor (Sibling = 10; Parent = 4) Matched Family donor with 9/10 bidirectional mismatch Matched Family donor with homozygous 8/10 in GvH direction Matched unrelated donor Matched unrelated donor with 9/10 homozygous allele in GvH direction	14 1 1 8 2
**Pre-transplant CMV status (recipient/donor)**	
pos/pos pos/neg neg/pos On IVIg/pos	19 3 2 2
**Pre-transplant EBV status (recipient/donor)**	
pos/pos pos/neg neg/pos neg/neg pos/not done neg/not done On IVIg/pos On IVIg/neg	13 1 1 1 4 4 1 1

*Cumulative dose of chemotherapy regimens: Treosulfan < 3 months = 30 gm/m^2^ × 3, 4 to 12 months = 36 gm/m^2^, > 1 year = 42 gm/m^2^; Fludarabine = 160 mg/m^2^; Thiotepa = 10 mg/kg; Cyclophosphamide = 120 mg/kg for acquired aplastic anemia; Cyclophosphamide = 40 mg/kg for Fanconi anemia.

### Graft characteristics and immune-reconstitution

**Figure 1:**
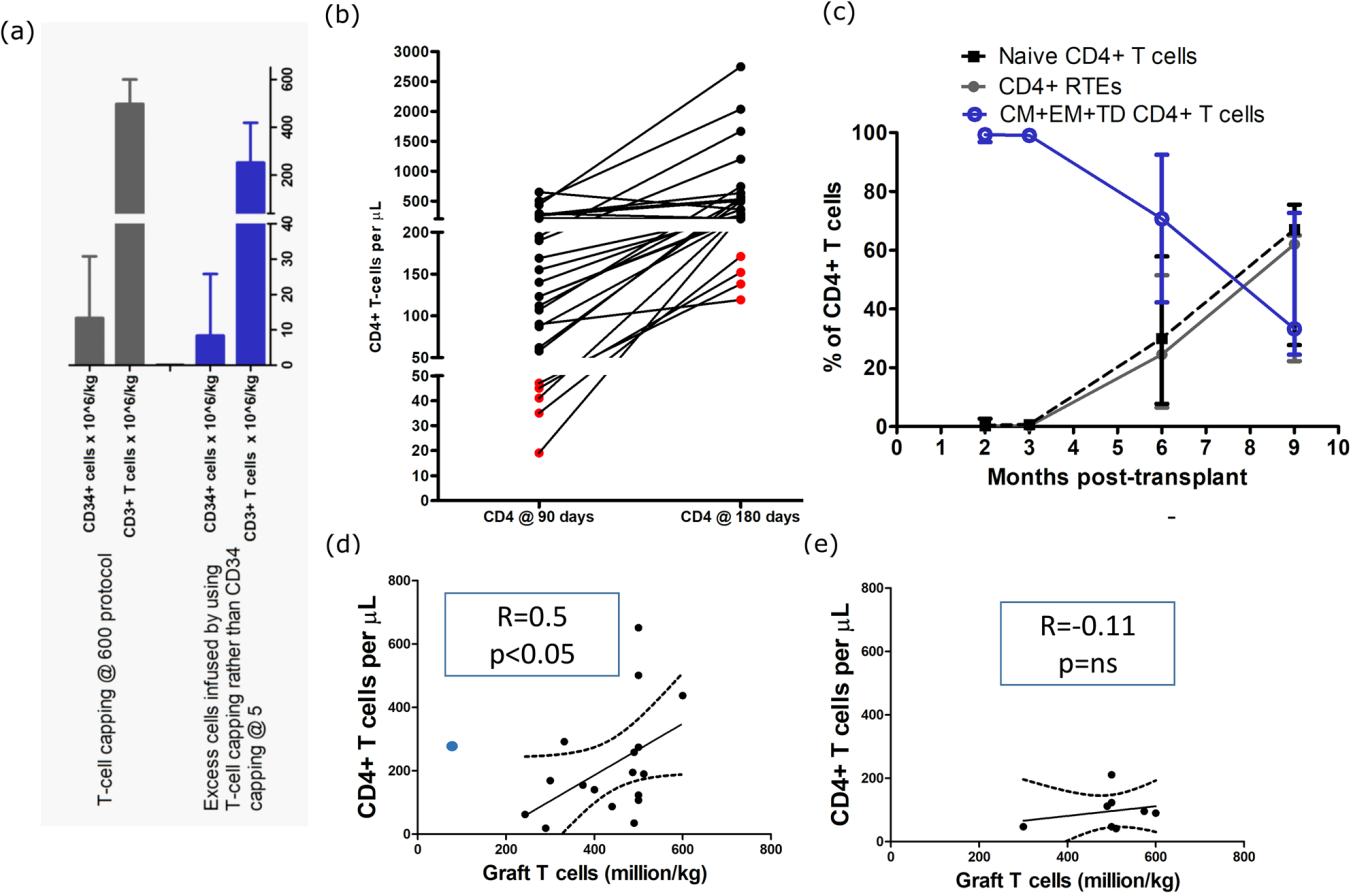
(**a**) CD34 + cell and CD3 + T-cell content in the graft using capping of T cells to a maximum T cells of 600 million/kg of recipient weight is shown. Excess CD34 + cells and T cells grafted using T-cell capping method rather than CD34 + capping at 5 million/kg are also shown. 1 (**b**) The primary outcome measure of CD4+ T-cell reconstitution at day + 90 and day + 180 is shown. The patients who did not meet the outcome measure are shown in red, and the connecting lines between day + 90 and day + 180 are shown to indicate the trend of CD4+ T-cell reconstitution. 1 (**c**) Percent of naïve versus central memory + effector memory + terminal differentiated (CM + EM + TD) CD4+ T cells and percent of CD4+ recent thymic emigrants (RTEs) in 4 children are shown. Circulating naïve CD4+ T cells and RTEs were absent at 3 months post-transplant confirming the differential effect of alemtuzumab on naïve and memory-effector-terminal differentiated subsets. Naïve T-cell reconstitution at 6 and 9 months correlated with the emergence of RTEs suggesting naïve T-cell reconstitution is via thymic output. 1 (**d**) Correlation between graft T-cell dose and CD4 reconstitution (at 90 days) in 18 patients who did not receive steroids is shown. A direct correlation was observed in patients not receiving steroids excluding the one outlier (a patient with aplastic anemia shown in blue) that developed early mixed chimerism and had received lowest T-cell dose of 90 million/kg. 1 (**e**) There was no correlation between graft T-cell dose and CD4 recovery in 8 patients receiving steroids. Seven patients were treated with a short course of systemic steroids (one to three weeks) for engraftment syndrome (n = 1); autoimmunity (n = 1) and acute GvHD (grade 1 skin; n = 5). One patient with acute GvHD (skin and gut; grade 3) required immunosuppression (including steroids, etanercept and ruxolitinib) for a total of 458 days.

The median T-cell dose was 500 million/kg (range: 90–600) with a CD34+ cell dose of 13.37 million/kg (6.74–30.8) (Fig. 1 a). With the capping of T cells rather than capping of CD34+ cells at 5 million/kg, we were able to graft with an additional 252 million/kg (29.8–418.8 million/kg) of T cells and 8.37 million/kg (1.74 –25.8 million/kg) of CD34+ cells (Fig. 1 a).

All patients had neutrophil and platelet engraftment. Neutrophil and platelet engraftment was at a median of 13 days (10–42) and 10 days (7–109) respectively.

The primary outcome measures of CD4+ T-cell recovery of 1) 50/µL at 90 days (−/ + 10) was met in 81% (21/26) patients and 2) 200/µL at 180 days (−/ + 10) was met in 85% (22/26) patients. The median number of CD4+ T cells was 131/µL (19–651/µL) at 90 days and 324/µL (119–2748) at 180 days. (Fig. 1 b). The median CD3 + T-cells at 90 days were 653/µL (75–1710/µL) and at 180 days were 1097/µL (476–3855/µL) (Supplemental figure S1).

We performed an extended phenotypic analysis in four children to understand the mechanism of early T-cell reconstitution. There was a near absence of naïve T cells in the first 3 months, and most early recovering cells were central memory and effector cells. The reconstitution of naïve T cells was observed with the onset of thymopoiesis at 6 months as confirmed by the serial measurement of naïve T cells and recent thymic emigrants (Fig. 1 c; supplemental figure S2).

We also analyzed the correlation between T-cell dose and CD4+ T-cell reconstitution. In 18 patients who did not receive systemic steroids after transplant, we observed a positive correlation between T-cell dose and CD4+ T-cell recovery at 90 days, excluding the one outlier; a patient with aplastic anemia who developed early mixed chimerism and had received the lowest T-cell dose of 90 million/kg (*p* < 0.02, r = 0.50; Fig. 1d). There was no correlation between T-cell dose and CD4+ T-cell recovery in patients who received systemic steroids (Fig. 1 e).

### Graft-versus-host disease

**Figure 2:**
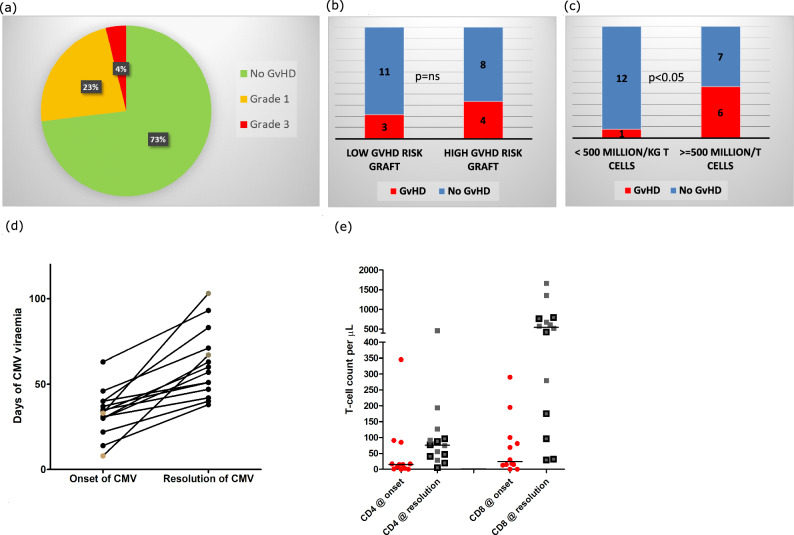
(**a**) Incidence and Grade of GvHD in all patients. 2 (**b**) GvHD incidence and grade in patients with low-GvHD risk graft versus high-GvHD risk graft is shown. No statistically significant difference was seen. 2 (**c**) GvHD incidence was high in patients receiving ≥ 500 million T cells compared to those who received < 500 million T cells. 2 (**d**) Days after transplant when onset and resolution of CMV viraemia occurred is shown. The majority of patients had predictable CMV viraemia with less than 30 days of viraemia. Two patients shown in grey dots had prolonged viraemia. 2 (**e**) CD4+ and CD8 + T cells at the onset and resolution of CMV viraemia are shown. Patients receiving high dose alemtuzumab are shown as squares with black borders. CD4+ and CD8 + T-cell responses were observed at the resolution of viraemia irrespective of the dose of alemtuzumab.

Despite the early CD4+ immune-reconstitution, only one patient had severe acute GvHD (grade 3, skin and gut), and six patients had grade 1 GvHD involving skin (Fig. 2 a). There was no statistical difference in the incidence of GvHD in patients receiving low GvHD risk graft versus those receiving high GvHD risk graft (Fig. 2b). Interestingly, forty-six percent of patients receiving 500 million/kg or more T cells developed GvHD, whereas only 8% of patients receiving less than 500 million/kg of T cells developed GvHD (*p* < 0.05; Fig. 2c). A patient who developed grade 3 GvHD received 600 million/kg T cells from a matched family donor. These observations indicate the role for controlling the T-cell dose in patients receiving alemtuzumab serotherapy.

Immunosuppression was weaned early in 15 patients due to the following reasons: 1) Mixed chimerism (n = 9), viral reactivation (n = 3), disseminated scabies (n = 1), drug-induced thrombotic-microangiopathy (n = 1), refractory autoimmune thrombocytopenia (n = 1). Out of the remaining eleven patients, immunosuppression was weaned in 9 patients at 6 months and in the 2 patients, immunosuppression was continued for primary disease-related inflammatory bowel disease and steroid-refractory GvHD. The median time to cessation of immunosuppression was 150 days (28–458 days). Despite the early cessation of immunosuppression, there was no evidence of chronic GvHD in any of the patient.

### Viral reactivation

CMV viraemia, adenoviraemia and EBV viraemia were seen in 14 (54%), 5 (19%) and 2 (8%) patients respectively. The treatment was given to 13 and 3 patients with CMV viraemia and adenoviraemia respectively. No patient with EBV viraemia required treatment.

CMV viraemia was studied in detail. The onset and resolution of CMV viraemia were at a median of 33 days (8–63) and 58 days (38–103) respectively (Fig. 2 d). CMV viraemia had a predictable duration of less than 30 days in 11 patients, and no patient had recurrence of CMV viraemia. CMV viraemia was treated with valganciclovir (n = 4), ganciclovir (n = 2), foscarnet (n = 6) or cidofovir (n = 1).

We measured CD4+ and CD8 + T cells at the time of onset and resolution of CMV viraemia. There was a significant difference in the number of circulating T cells at the onset and resolution of CMV viraemia. On comparing the T cells at the onset and resolution of CMV viraemia, both CD4+ T cells and CD8 + T cells were significantly higher at the resolution of CMV viraemia (*p* < 0.05; Fig. 2 e). The T-cell responses were observed before 90 days of transplant i.e. before the onset of thymopoiesis.

### Outcomes

Overall survival at one year was 100% and disease-free survival at a median follow-up of 899 days was 96% (Supplemental figure S3). One child with CTLA4 deficiency and severe inflammatory bowel disease showed initial improvement followed by recurrence of symptoms despite 100% donor chimerism, hence event-free survival at one year was 96%.

At last follow up, chimerism was 100% in 17 patients and mixed in 9 patients. Median chimerism in patients with mixed chimerism was 71% (40–98) and all patients with mixed chimerism were disease-free.

### Adverse events

Twenty-four adverse events were reported—23 were grade 3, 1 was grade 4, and no graft rejection or transplant-related mortality was recorded (Supplemental table S1). Infections and immunological disorders were observed in 22 patients, accounting for 92% of the total grade 3 or 4 adverse events. There were no serious adverse events related to alemtuzumab infusion.

## Discussion

Immune-reconstitution in transplant protocols utilizing serotherapy as the primary GvHD prophylaxis is determined by the two potential factors (1) serotherapy exposure of the graft and (2) T-cell content in the graft. Serotherapy exposure of the graft correlates inversely with the CD4+ T-cell reconstitution. Hence, Boelens et al. devised a study of individualised ATG dosing based on the quality of lymphocytes (cord blood versus bone marrow or PBSC donor), weight, and lymphocyte count of the host^5^. Using the individualised ATG dosing, the authors showed improved CD4+ T-cell reconstitution. In the context of alemtuzumab though, previous attempts of optimisation such as different doses and different timings of alemtuzumab in relation to the graft have not been successful in improving immune-reconstitution^6–8,10–12,21–27^.

The data from European centres reported to date show a late T-cell recovery in patients with alemtuzumab serotherapy^9–12,21^. Ottaviano et al^8^ reported a study of children with non-malignant disorders receiving peripheral blood stem cell transplant from five European centres over a period of 10 years. The incidence of GvHD in the alemtuzumab cohort was low but there was delayed T-cell immune-reconstitution in the first 3 months. Delayed T-cell recovery was significantly associated with mortality in the cohort. Another cohort of children reported by Willemsen et al^22^ had delayed T-cell recovery with alemtuzumab in both malignant and non-malignant disorders resulting in lower overall and event-free survival. Therefore to improve CD4+ T-cell immune-reconstitution, the precision dosing study of alemtuzumab in pediatric HSCT patients is underway (NCT05501756). We applied a different approach to the precision dosing study and showed that T-cell capping protocol facilitates early immune-reconstitution without increasing the risk of severe GvHD.

In our study of determining graft size based on T-cell content, we observed rapid recovery of CD4+ T cells at the early and late primary outcome measures. The early T-cell reconstitution i.e. homeostatic proliferation derived from the T cells carried with the graft in the first six months was rapid with the T-cell capping protocol in comparison to any of the previously published studies (Supplemental table S2)^6–8,23–27^. CD4+ T-cell recovery in the study is also comparable to the individualised ATG dosing study^5^. Interestingly, we found a correlation between T-cell dose and the occurrence of GvHD. There was no correlation between the occurrence of GvHD and the alemtuzumab dose, despite the inverse correlation between the alemtuzumab dose and T-cell recovery (data not shown). This suggests that a higher dose of alemtuzumab may be necessary for GvHD-risk grafts.  The correlation between T-cell dose and GvHD was striking. However, the incidence of severe acute GvHD was low and we did not observe any chronic GvHD despite the high numbers of graft T cells. The low incidence of severe acute and chronic GvHD may be because of depletion of naïve T cells and relative sparing of less alloreactive memory-effector T cells^28,29^.

The alemtuzumab-spared T cells may have also contributed to the clearance of viruses. The majority of transplant recipients were CMV seropositive and hence CMV was the model virus to study antigen-specific immune-reconstitution. CMV reactivation occurred only in half the children and the majority of patients with CMV reactivation had the predictable course of viraemia without recurrence of CMV. The resolution of viraemia was associated with CD4+ and CD8 + T-cell responses before the onset of thymopoiesis. The observations thus indicate the role of alemtuzumab-spared T cells in mediating the anti-CMV effect. It is therefore plausible that the relative sparing of memory and effector T cells following alemtuzumab serotherapy could facilitate the antigen-specific immune-reconstitution.

We also did not observe graft rejection in our cohort. The majority of previous studies have reported graft rejection in a proportion of patients receiving alemtuzumab serotherapy^6–8,23–27^. A recent study also highlighted the correlation between excessive depletion of graft T cells due to high alemtuzumab levels and increased risk of graft failure^4^. It is therefore conceivable that, as hypothesized, some T cells may escape alemtuzumab in patients receiving high numbers of graft T cells, thereby reducing the risk of primary or secondary graft rejection. 

The study is the first to show that T-cell capping is one of the ways of enhancing immune-reconstitution in patients receiving alemtuzumab serotherapy and PBSCs from HLA-matched donors. A correlation between T-cell dose versus CD4+ recovery and GvHD was observed, and thus these observations indicate the role for controlling the T-cell dose in patients receiving alemtuzumab serotherapy. Based on the observations in our study, we cap T cells at 500 million per kg of recipient weight with a minimum of 200 million per kg of recipient weight, as shown in the schematic model (Supplemental figure S4). A prospective observational study of day zero alemtuzumab levels is planned to determine the T-cell graft content relative to the alemtuzumab levels, and thus achieve predictable immune-reconstitution. However, there are possible limitations to the method of T-cell capping. T-cell capping may not be applicable for grafts with low T cells per kilogram of the recipient weight for eg. bone marrow and hence, its use will be limited to paediatrics and PBSC grafts. The utilization of PBSC grafts is increasing though because of ease of collection; hence this grafting technique holds the potential to become applicable globally^30,31^.

Although, the study has a limited number of patients and PBSC is not the preferred stem cell source in paediatric patients, the current study provides proof-of-concept for controlling the T-cell dose in patients receiving PBSC graft with alemtuzumab serotherapy. In conclusion, the T-cell capping protocol with alemtuzumab serotherapy is safe and leads to early T-cell immune-reconstitution without chronic GvHD.

### Supplementary Information

Supplementary Information.

## Data Availability

The datasets generated and/or analysed during the current study are not publicly available because patients and/or legal guardians have not consented to data sharing but are available from the corresponding author on reasonable request.
