# Supplementary material for: The role of graft T-cell size in patients receiving alemtuzumab serotherapy for non-malignant disorders: results of an institutional protocol
Source: Sci Rep. 2024 Jan 10;14:988. doi: 10.1038/s41598-023-50416-6 (PMC10781954; doi:10.1038/s41598-023-50416-6)
Supplement: Supplementary file 1 — Supplementary Information. [file 41598_2023_50416_MOESM1_ESM.pdf]

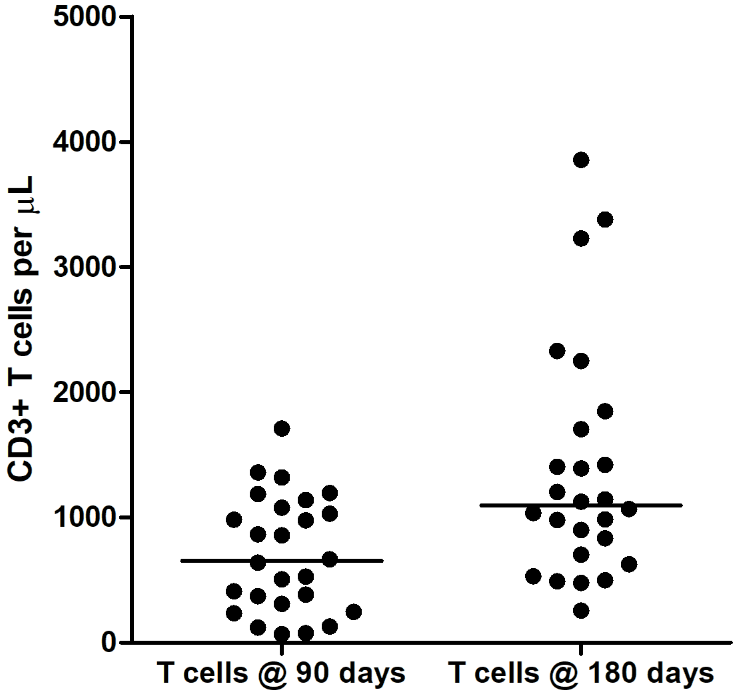

Supplemental figure 1 CD3+ T cells at day+90 and day+180 after transplant are shown.

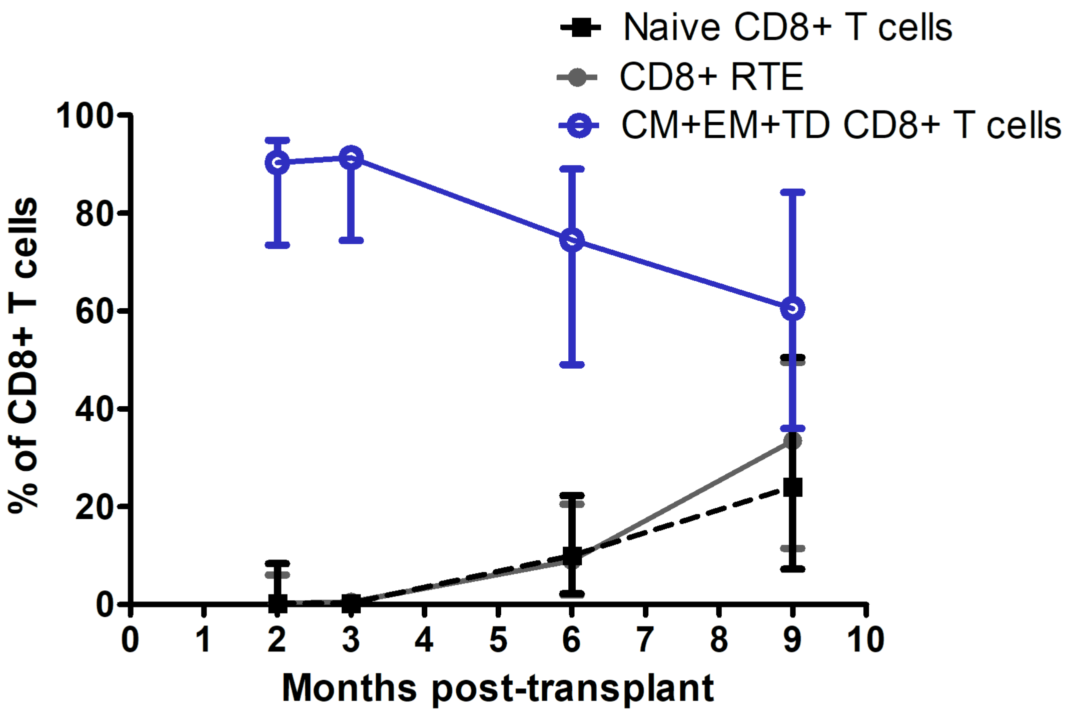

Supplemental figure 2 Reconstitution of naive CD8+ T cells, CD8+ RTE's and CD8+ memory-effector T cells are shown.

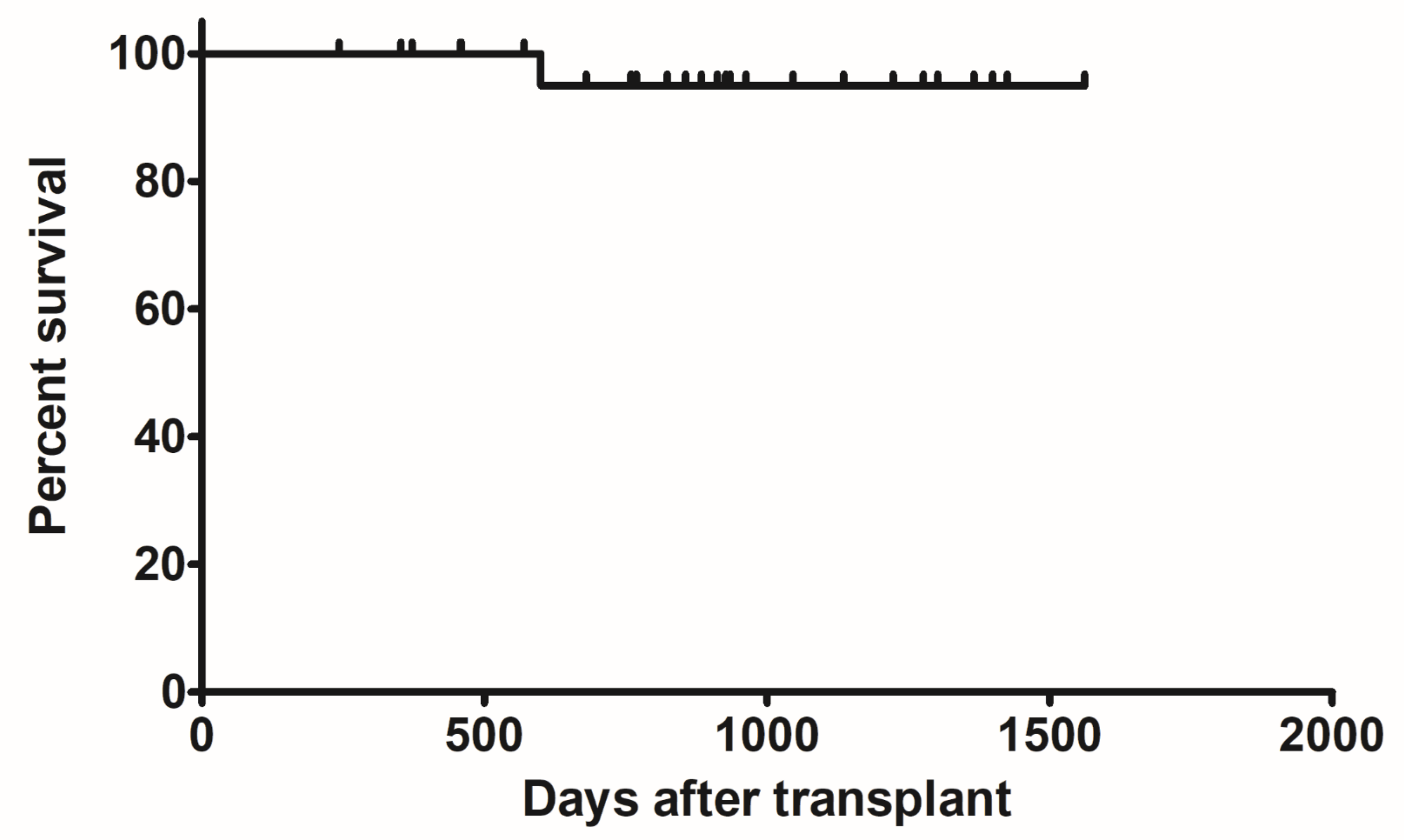

Supplemental figure 3 Event-free survival

CD34 capping @ 5 million/kg

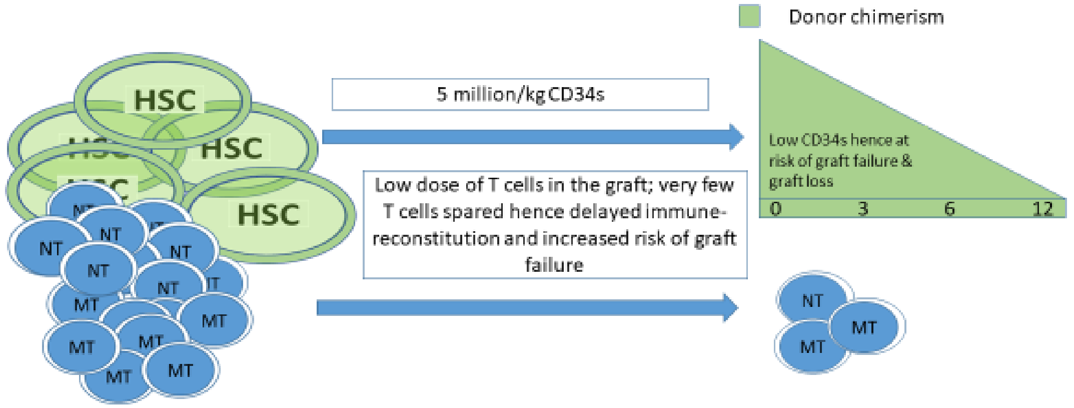

T-cell capping @ 500 million/kg

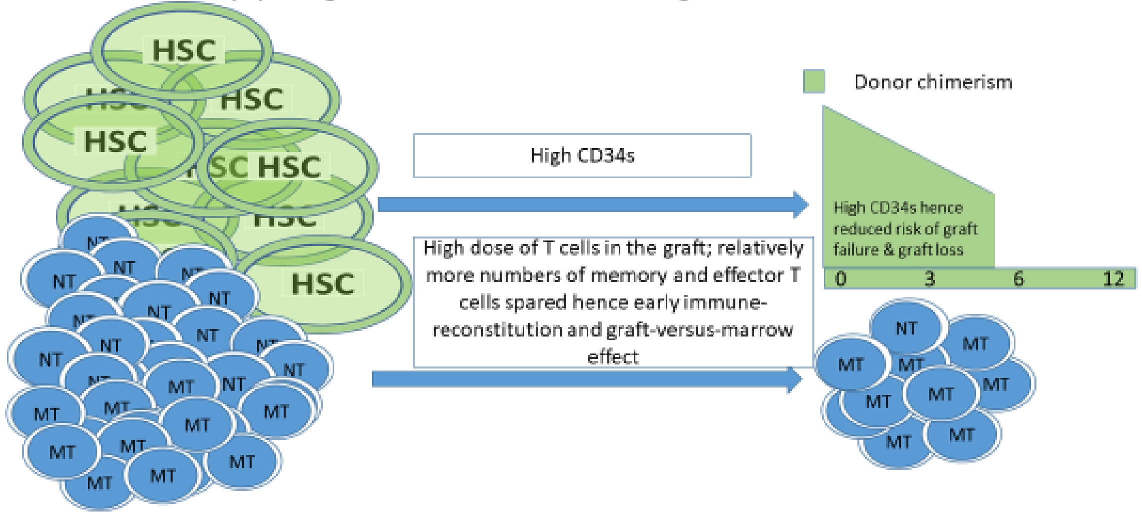

Supplemental figure 4 a Schematic model of grafting with conventional CD34+ capping protocol and alemtuzumab serotherapy
Abbreviations: HSC – hematopoietic stem cells; NT – naïve T cells; MT – memory and effector T cells

Supplemental figure 4 b Schematic model of grafting with T-cell capping protocol and alemtuzumab serotherapy
Abbreviations: HSC – hematopoietic stem cells; NT – naïve T cells; MT – memory and effector T cells

Supplemental table 1 Overview of adverse events

	Grade 3	Grade 4
**Infections**		
BK virus cystitis	0	0
Candida sepsis	0	0
Catheter-related infection	0	0
Cytomegalovirus reactivation	5 (19%)	0
Adenovirus reactivation	3 (12%)	0
Parvoviraemia	0	0
Epstein-Barr virus reactivation	0	0
Febrile neutropenia	5 (23%)	0
Fungal pneumonia	0	0
Influenza	0	0
Lung infection	1 (4.5%)	0
Pneumocystis jirovecii pneumonia	0	0
Sepsis	1 (4.5)	0
Skin infection	0	0
Upper respiratory tract infection	0	0
Urinary tract infection	0	0
Viral gastro-enteritis	0	0
**Immune system disorders**		
Allergic reaction (to alemtuzumab)	0	0
Allergic reaction (to liposomal amphotericin B)	0	0
Auto-immune cytopenia	3 (13.6%)	0
Auto-immune thrombocytopenia	1 (4.5%)	0
Engraftment syndrome	1 (4.5%)	0
Capillary leak syndrome	0	0
Cytokine release syndrome	0	0
Graft failure	0	0
Graft-versus-host disease	1 (4.5%)	0
Immune dysregulation	0	0

Progression of underlying disease (lung disease, immunodeficiency, chromosomal breakage syndrome)	1 (4.5%)	0
Secondary haemophagocytic lymphohistiocytosis	0	0
**Gastro-intestinal**		0
Dehydration	0	0
Feeding problems	0	0
Gastro-intestinal haemmorrhage	0	0
Gastro-oesophageal reflux disease	0	0
Pancreatitis	1 (4.5%)	
**Metabolism and nutrition**		0
Hypokalemia	0	0
**Cardiac disorders**		
Pericardial effusion	0	1 (4.5%)
**Hepatobiliary disorders**		
Hyperbilirubinaemia	0	0
Veno-occlusive disease	0	0
**Respiratory, thoracic, and mediastinal disorders**	0	0
Bronchiolitis obliterans	0	0
**Nervous system disorders**		
Seizure	0	0
Neuralgia	0	0
**Renal and urinary disorders**		
Acute kidney injury	0	0
TA-TMA	0	0

Supplemental table 2 Literature review of alemtuzumab use in non-malignant disorders

Author	No. of patients	Disease condition	Timing of alemtuzumab	Donor	Stem cell source	Median CD34+/T-cell dose (million per kg)	GVHD Acute (Grade & %)	GVHD Chronic (Limited/ Extensive)	Immune-reconstitution	Viral reactivation CMV ADV	Rejection and intervention for falling chimerism	Transplant-related mortality	Disease-free/ Overall survival (%)
Mahadeo[1] 2014 (prospective pilot)	22	NMDs	not mentioned	MSD	not mentioned	7.6/NA	No	No	CD3 500/µL @ day+174	5/22 recipients (45% were donor/recipient neg/neg)<br>1/22 ADV	2 patients were given DLI for falling chimerism	9%	90%/90%
Oshrine[2] 2014 (retrospective)	31	NMDs	day- 21 or day-13 or day-9	14 MSD<br>16 MUD<br>1 MMRD	100% BM	NA/NA	Grade 3-4 16.1%	Limited & Extensive 26.1%	CD3 197/µL in day-9/-13 vs 668/ µL in day-21 @ 4 months	22.6% CMV (CMV serostatus not mentioned)<br>12.9% ADV	6/27 (22.2%)<br><br>6 patients – DLI<br>4 patients – 2nd graft	9.6%	65.9%/85.3%
Saif[3] 2015 (retrospective)	91	NMDs	day-9 to day-5 or<br>day-7 to day-5	59 MUD<br>32 MRD	58% BM<br>38% PBSC	NA/NA	Grade 2-4 18.7%	Extensive 5.5%	Median time CD4 recovery = 11 months (4 – 48)<br>CD8 recovery = 6 months (1 – 26)	CMV 39.6% (46% were donor/recipient neg/neg)<br>ADV 19.8%	9/91 (9.9%)<br>9 patients – 2nd graft	8.8%	91.2%/91.2%
Abdel-Azim[4] 2015 (prospective pilot)	15	NMDs	day-10 to day-8	15 MUD	100% BM	6/NA	Grade 1-2 46.6%<br>Grade 3-4 0%	Extensive 6.6%	Median days T-cell > 30/µL @ day+92 (50 – 282)	CMV 0% (all received Ganciclovir till day-2; 40% were donor/recipient neg/neg)<br>ADV 20%	3/15 (20%)<br>2 patients – 2nd graft	6.6%	73.3%/93.3%
Marsh[5] 2015 (retrospective)	206	NMDs	30% intermed<br>36% distal<br>28% proximal<br>7% other	38 MRD<br>115 MUD<br>57 MMD	90% BM<br>5% CB<br>5% PBSC	5.3/NA	Grade 2-4 25%<br>Grade 3-4 18%	Extensive 11%	NA	NA	10/210 (5%)<br>46 patients – stem cell product or DLI<br>10 – 2nd graft	28.6%	OS - 78%
Bhatt[6] 2019 (prospective pilot)	71	NMDs	day-21 to day-19	39 MRD<br>32 MUD	86% BM<br>11% PBSC<br>3% BM+CB	NA/NA	Grade 3-4 19.7%	Extensive 16.9%	@ 100 days<br>Mean CD3 ~ 500/ µL in MRD and 250/µL in MUD	CMV 22.5% (39.4 were donor/recipient neg/neg)<br>ADV 5.6%	9/71 (12.6%)	5.5%	not mentioned
Contreras[7] 2020 (retrospective)	62	NMDs	day-12 to day-10	12 MSD<br>17 MUD<br>2 MMRD<br>2 MMUD	70% BM<br>30% PBSC	NA/NA	Grade 3-4 3.2%	Extensive 1.6%	Median time to CD4 >200/µL & PHA>50% 181 days (76 – 585)	CMV 25.8% (46.7 recipients had negative serostatus)<br>ADV 9.7%	5/62 (8%)<br>2 patients – DLI	10%	80.3%/86.5%
Ottaviano[8] 2021 (retrospective)	63	NMDs	day-5 to day-1	52 MUD<br>2 MSD<br>9 MRD	100% PBSC	NA/NA	Grade 3-4 10%	Extensive 5%	CD3 @ 90 days = 150/µL	CMV 27%<br>ADV 23%	4/62 (6.4%)	13%	79%/85%
T-cell capping protocol	26	NMDs	day-6 to day-4	14 MRD (10/10)<br>1 MRD (9/10 bidirectional)<br>1 MRD (8/10 GvH)<br>8 MUD (10/10)<br>2 MUD (9/10 GvH)	100% PBSC	13/500	Grade 3-4 4%	None	CD3 @ 90 days = 653 µL<br>Median CD4 @ 90 and 180 days = 131/µL & 324/µL respectively	CMV 59%<br>ADV 14%	0	0%	95%/100%

Abbreviations: NMDs – non-malignant disorders, MSD –matched sibling donor, MUD – matched unrelated donor, MRD – matched related donor, MMRD – mismatched related donor, MMUD – mismatched unrelated donor, BM – bone marrow, CB – cord blood, PBSC – peripheral blood stem cells, NA – not available, CMV – cytomegalovirus, ADV – adenovirus, OS – overall survival

References

1. Kris Michael Mahadeo, Rajni Agarwal, Kenneth I. Weinberg, Hisham Abdel-Azim, David B. Miklos, Ami J. Shah, Laraib Tabba, Neena Kapoor. Durable Engraftment, Correction of Genetic Defects and Prevention of Veno-Occlussive Disease, Following Blood and Marrow Transplantation with an HLA-Matched Sibling DONOR, Using a Reduced Toxicity Conditioning Regimen with Busulfan, Reduced Dose Cyclophos, Biology of Blood and Marrow Transplantation,Volume 20, Issue 2, Supplement,2014, Page S83. ISSN 1083-8791.
2. Oshrine BR, Olson TS, Bunin N. Mixed chimerism and graft loss in pediatric recipients of an alemtuzumab-based reduced-intensity conditioning regimen for non-malignant disease. Pediatr Blood Cancer. 2014 Oct;61(10):1852-9.
3. Saif MA, Borrill R, Bigger BW, Lee H, Logan A, Poulton K, Hughes S, Turner AJ, Bonney DK, Wynn RF. In vivo T-cell depletion using alemtuzumab in family and unrelated donor transplantation for pediatric non-malignant disease achieves engraftment with low incidence of graft vs. host disease. Pediatr Transplant. 2015 Mar;19(2):211-8.
4. Abdel-Azim H, Mahadeo KM, Zhao Q, Khazal S, Kohn DB, Crooks GM, Shah AJ, Kapoor N. Unrelated donor hematopoietic stem cell transplantation for the treatment of non-malignant genetic diseases: An alemtuzumab based regimen is associated with cure of clinical disease; earlier clearance of alemtuzumab may be associated with graft rejection. Am J Hematol. 2015 Nov;90(11):1021-6.
5. Marsh RA, Rao MB, Gefen A, Bellman D, Mehta PA, et al. Experience with Alemtuzumab, Fludarabine, and Melphalan Reduced-Intensity Conditioning Hematopoietic Cell Transplantation in Patients with Nonmalignant Diseases Reveals Good Outcomes and That the Risk of Mixed Chimerism Depends on Underlying Disease, Stem Cell Source, and Alemtuzumab Regimen. Biol Blood Marrow Transplant. 2015 Aug;21(8):1460-70.
6. Bhatt ST, Schulz G, Hente M, Slater A, Murray L, Shenoy S, Bednarski JJ. A single- center experience using alemtuzumab, fludarabine, melphalan, and thiotepa as conditioning for transplantation in pediatric patients with chronic granulomatous disease. Pediatr Blood Cancer. 2020 Jan;67(1):e28030. doi: 10.1002/pbc.28030.
7. Contreras CF, Long-Boyle JR, Shimano KA, Melton A, Kharbanda S, Dara J, Higham C, Huang JN, Cowan MJ, Dvorak CC. Reduced Toxicity Conditioning for Nonmalignant Hematopoietic Cell Transplants. Biol Blood Marrow Transplant. 2020 Sep;26(9):1646-1654.
8. Ottaviano G, Achini-Gutzwiller F, Kalwak K, Lanino E, Faraci M, Rao K, Chiesa R, Amrolia P, Bonanomi S, Rovelli A, Veys P, Lankester A, Balduzzi A, Lucchini G. Impact of in Vivo Lymphodepletion on Outcome in Children with Nonmalignant Disorders Receiving Peripheral Blood Stem Cell Transplantation. Transplant Cell Ther. 2021 Dec;27(12):1020.e1-1020.e5.
